# Can Multifrequency Tympanometry Be Used in the Diagnosis of Meniere’s Disease? A Systematic Review and Meta-Analysis

**DOI:** 10.3390/jcm13051476

**Published:** 2024-03-04

**Authors:** Christos Tsilivigkos, Evangelos N. Vitkos, Eleftherios Ferekidis, Athanasia Warnecke

**Affiliations:** 1 First Department of Otolaryngology, Hippokration General Hospital, National and Kapodistrian University of Athens, 15772 Athens, Greece; drferekidis@gmail.com; 2 Department of Oral and Maxillofacial Surgery, George Papanikolaou General Hospital, 56429 Thessaloniki, Greece; 3 Department of Otorhinolaryngology—Head and Neck Surgery, Hannover Medical School, 30625 Hanover, Germany; 4 Cluster of Excellence Hearing4all, German Research Foundation, 30625 Hannover, Germany

**Keywords:** multifrequency tympanometry, impedance tympanometry, tympanometry, Meniere’s disease, endolymphatic hydrops, systematic review, meta-analysis

## Abstract

**(1) Background**: Ménière’s disease (MD) is a disease of the inner ear, presenting with episodes of vertigo, hearing loss, and tinnitus.The aim of this study is to examine the role of multifrequency tympanometry (MFT) in the diagnosis of MD. **(2) Methods:** A systematic review of MEDLINE (via PubMed), Scopus, Google Scholar, and the Cochrane Library was performed, aligned with the PRISMA guidelines. Only studies that directly compare ears affected by Ménière’s disease with unaffected or control ears were included. Random-effects model meta-analyses were performed. **(3) Results:** Seven prospective case-control studies reported a total of 899 ears, 282 of which were affected by Ménière’s disease (affected ears—AE), 197 unaffected ears in patients with MD (UE), and 420 control ears (CE) in healthy controls. No statistically significant differences between the groups were observed regarding resonant frequency (RF). The pure tone audiometry average of the lower frequencies (PTA basic) was significantly greater in affected ears when compared with unaffected ears. The conductance tympanogram at 2 kHz revealed a statistically significantly greater G width of 2 kHz in the affected ears when compared to both unaffected and control ears, while control ears had a statistically significant lesser G width of 2 kHz compared to both the other two groups. **(4) Conclusions:** MFT, and specifically G width at 2 kHz, could be an important tool in the diagnosis of MD.

## 1. Introduction

The middle ear can be considered a delicate impedance-matching system, providing an effective way of sound transmission from the larger tympanic membrane and through the ossicular chain to the smaller oval window [1]. The mechanical journey of the sound continues into the cochlear liquids before mechano-electrical transduction takes place in the inner hair cells of the cochlea. This optimal sound energy transmission through the system lies in humans between 1 and 2 kHz, a range that corresponds to most of the speech cues contained in human speech [2].

Acoustic impedance is an important physical property of the tympano-ossicular system that expresses the opposition of the system to a wave of sound energy that passes through it. Acoustic impedance depends on the vibration properties of the medium [3]. As has been shown in early experiments by Terkildsen and Thomsen, changes in the pressure of the middle ear cavity impact the impedance of the system. Classic tympanometry was the technique initially used to evaluate these alterations in acoustic impedance due to pressure changes behind the tympanic membrane [3].

Tympanometry has evolved over the last decades and is now one of the most common audiological assessments applied worldwide [4]. Although it is an easy, sensitive, and fast procedure, a tympanogram only evaluates the system at a specific frequency of 226 Hz [5]. Pathologies affecting the ossicular chain, e.g., otosclerosis, are commonly misdiagnosed with classic tympanometry [6]. This limitation led to the development of multifrequency, multicomponent tympanometry (MFT), a technique that assesses multiple components of the acoustic immittance (multicomponent) through a large array of frequencies spanning from 226 to 2000 Hz (multifrequency) [6,7].

Meniere’s disease (MD) is a clinical condition with a prevalence of 50–200/100,000 adults and presents commonly in the age range of 40 to 60 years. The disease is considered to affect the inner ear, presenting clinically with fluctuating symptoms of vertigo, hearing loss, tinnitus, and aural fullness. Endolymphatic hydrops, a condition that increases pressure levels in the membranous labyrinth, is the pathognomonic finding of MD [8]. Specific diagnostic criteria for MD have been proposed by different audiology associations, suggesting pure tone audiometry as a significant test in the diagnostic battery of the disease [9,10].

According to the most recent guidelines, the diagnosis for MD is clinical and can be categorized as definite or probable. The following criteria are required for the diagnosis of definite MD:

1. At least two spontaneous episodes of vertigo with a duration of 20 min to 12 h.
2. Pure tone audiogram reveals sensorineural hearing impairment in low and mid frequencies in the affected ear before, during, and/or after one of the episodes.
3. Symptoms of hearing loss, tinnitus, and aural fullness in the affected ear present with fluctuation.
4. Not better accounted for by other vestibular pathologies [11].

Diagnosis of probable MD includes broader criteria regarding the episodes of vertigo or dizziness of the patient [11].

Several diagnostic tools to assess endolymphatic hydrops and their consequences in the cochlea have been proposed over the years, such as electrocochleography, which has been assessed in patients with MD since the 1970s [12]. Other relevant tools include vestibular evoked myogenic potentials (VEMPs) [13], the glycerol (Klockhoff’s) test, and fluid-attenuated inversion recovery of magnetic resonance imaging (3D-FLAIR-MRI) with contrast agents [14]. These, however, can be expensive and time-consuming procedures, and they need expertise.

On the other hand, MFT could be a time-sparing test used in the diagnosis of MD. As proposed in an experiment conducted on guinea pigs, injection of scala tympani with saline would increase the perilymphatic and, consequently, the endolymphatic pressure, thus leading to a double peak in the conductance (G) component at 2 kHz of MFT. The reverse experiment of removing liquid from the cochlea would result in a decrease in the cochlear pressure and the disappearance of the peaks. A further increase in the cochlear pressure is considered to augment the width between the two peaks in conduction tympanometry at 2 kHz. Overall, shifts in cochlear pressure and annular ligament caused by endolymphatic hydrops could result in changes in the admittance, conductance, and susceptance components at 2 kHz of MFT [15].

The need for a sensitive, cost-effective, and time-sparing technique to assist in the diagnosis of MD led us to conduct a systematic review and meta-analysis of the possible application of MFT. To our knowledge, this is the first paper addressing this issue in terms of a systematic review and meta-analysis. We conducted a comprehensive synthesis of all available data to highlight the possible role of MFT as a diagnostic tool in MD.

## 2. Materials and Methods

### 2.1. Protocol and Registration

The protocol of the current research is registered a priori in the international PROSPERO under the reference number CRD42023438284.

### 2.2. Study Design

To address this research question, a systematic review of the literature and meta-analysis according to the PRISMA (Preferred Reporting Items for Systematic Reviews and Meta-Analyses) statement was reported [16]. We defined our research question by applying the PICO (Population/Participants, Intervention, Comparison, Outcome) framework:

Population/Participants: Adult (>18 years old) patients with Meniere’s disease (affected ears and unaffected ears) who underwent multifrequency tympanometry assessment.

Intervention: Multifrequency tympanometry and pure tone audiometry in affected ears.

Comparison: Multifrequency tympanometry and pure tone audiometry in control or unaffected ears.

Outcomes: The primary outcomes assessed were the pure tone audiometry average of the lower frequencies (PTA basic), resonant frequency (RF), G width of 2 kHz, and Y width of 2 kHz.

### 2.3. Eligibility Criteria

All studies published in the English language that reported outcomes from multifrequency tympanometry in adults with at least one ear suffering from Ménière’s disease, of any age, sex, or ethnicity, and directly compared them with unaffected or control ears were included in the study. Ears diagnosed with MD were categorized as affected ears, while ears without the pathology of MD in patients with unilateral MD were categorized as unaffected ears. Ears in healthy controls with both ears unaffected by MD were categorized as control ears. All ears, affected, unaffected, or controlled, should be free of middle ear disease and have an intact tympanic membrane.

We excluded multiple studies according to the following criteria: (i) studies that were published in languages other than English; (ii) case reports or case series; (iii) systematic reviews of interventions or meta-analyses; (iv) studies that reported outcomes without comparator groups; (v) theses; (vi) studies with less than five reported patients per each group; (vii) studies with irrelevant interventions (e.g., classic tympanometry, wideband tympanometry, electrocochleography, VEMPs) or patient groups (e.g., patients with middle ear diseases or other inner ear pathology); (viii) studies including patients during an acute episode of MD. We agreed a priori that in cases where multiple studies report the same population, the study with the best design would be included in our meta-analysis. No publication-year limitation was applied.

### 2.4. Search Strategy

We searched Medline via PubMed using the algorithms (“multifrequency tympanometry” OR “multicomponent tympanometry” OR “multicomponent multifrequency tympanometry”) AND (“meniere” OR “meniere disease” OR “meniere disease”[MeSH Terms] OR “labyrinth Diseases” OR “labyrinth Diseases”[MeSH Terms]), Cochrane Database, Google Scholar, and Scopus with relevant algorithms with a search date of 25 February 2023. Two independent reviewers (CT and ENV) screened the title and abstract of the resulting articles and assessed them for eligibility. Then, a full-text evaluation was performed on the selected articles to finalize the included ones by the same authors. In the event of disagreement between the authors, a consensus would be reached after discussion with the senior author (AW). The references to the included studies were also searched manually for any potentially eligible study [17].

### 2.5. Data Extraction and Tabulation

The first author (CT) and the second author (ENV) worked independently and extracted the data from the included studies into a standardized, pre-designed formula for evidence collection. All potential disagreements were resolved after reaching a consensus with the senior author (AW). The data extracted were the following: (i) study characteristics (authors, year, country), the total number of ears, ears suffering Ménière’s disease (affected ears), unaffected ears, control ears, (ii) patient’s baseline characteristics (age and sex), (iii) tympanometry outcomes (RF, G width 2 kHz, and Y width 2 kHz), and the pure tone audiometry average of the lower frequencies (PTA basic). We used only each study’s available data.

### 2.6. Quality of Evidence Assessment

All the included studies in our systematic review were non-randomized prospective cohort studies. We assessed their quality using the Newcastle Ottawa Scale (NOS) to assess the risk of bias in non-randomized studies of interventions [18]. Two independent researchers (CT and ENV) applied the tool to each of the included studies and examined the nine domains that NOS addresses. Six of the seven included studies were assessed as high quality and one as fair (Supplemental Material Table S1).

### 2.7. Statistical Analysis

We performed a meta-analysis to compare the outcomes of tympanometry in ears affected by Ménière’s disease when compared to unaffected or control ears, as well as the outcomes of tympanometry between unaffected and control ears. The variables examined were continuous and are presented as means and standard deviations. We converted the data given as median and range to mean and standard deviation using the Hozo et al. method [19]. They were analyzed using the mean difference (Mdiff) and 95% confidence intervals (CI). The inherent clinical heterogeneity between the studies was balanced using the random effects models (DerSimonian–Laird). Forest plots were generated to display the results. The between-study statistical heterogeneity was assessed with Cochran’s Q statistic and by estimating I^2^. High heterogeneity was confirmed with a significance level of *p* < 0.10 and I^2^ ≥ 50%. Publication bias was considered statistically significant in cases of *p* < 0.10. All analyses were performed using RSTudio 4.2.1.

## 3. Results

### 3.1. Study Selection and Baseline Characteristics

**Figure 1:**
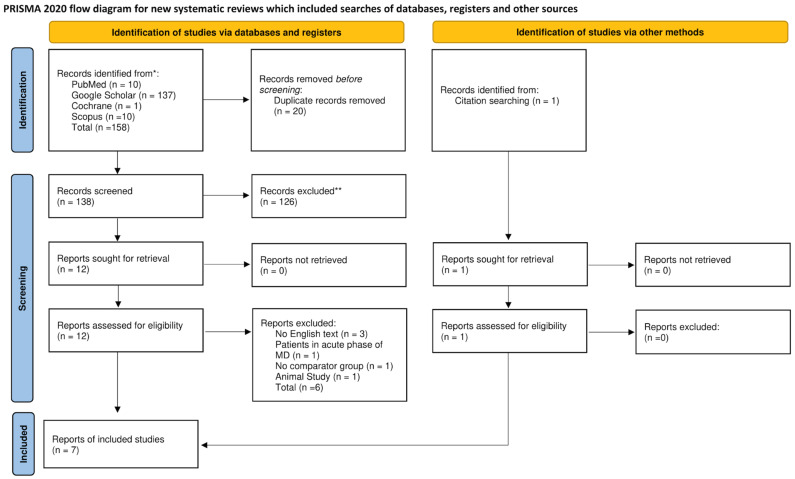
PRISMA flowchart. * Consider, if feasible to do so, reporting the number of records identified from each database or register searched (rather than the total number across all databases/registers). ** If automation tools were used, indicate how many records were excluded by a human and how many were excluded by automation tools. From: Page MJ, McKenzie JE, Bossuyt PM, Boutron I, Hoffmann TC, Mulrow CD, et al. The PRISMA 2020 statement: an updated guideline for reporting systematic reviews. BMJ 2021;372:n71. doi: 10.1136/bmj.n71 [16]. For more information, visit: http://www.prisma-statement.org/.

After performing a systematic literature search, we retrieved a total of 158 articles. The duplicate articles were 20 and therefore excluded. In the remaining 138 articles, we performed title and abstract screening, which led to the exclusion of 126 more articles, and the remaining 12 studies were deemed eligible for full-text review. Through full-text review, we excluded six more studies, three of them due to lacking an English text, one due to reporting patients during an acute phase of MD, one due to no comparator group, and one animal study [20,21,22,23,24,25]. One additional article was identified through the manual search of the references to the retrieved articles [26]. The literature search is illustrated in the PRISMA flowchart (Figure 1).

Ultimately, seven studies reported a total of 899 ears, 282 of which were affected by Ménière’s disease, 197 were unaffected ears, and 420 were control ears. Four of them were conducted in Japan [26,27,28,29], one of them in France [30], and one in Turkey [31] and the Netherlands, respectively [32]. All seven studies had a prospective cohort design. We present the baseline characteristics of the included studies and the reported patients in Table 1. The summary of our analysis is presented in Table 2.

### 3.2. Outcomes

#### 3.2.1. G Width 2 kHz

**Figure 2:**
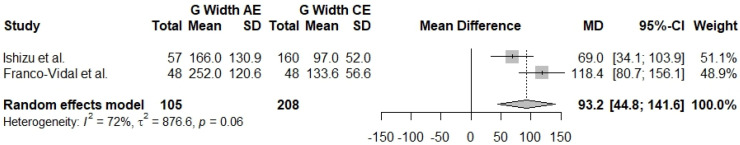
Forest plot comparing G width at 2 kHz between affected ears (AE) and control ears (CE) [27,30].

**Figure 3:**
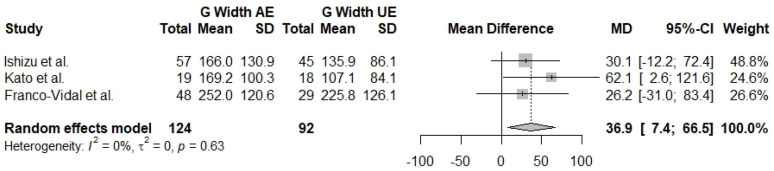
Forest plot comparing G width at 2 kHz between affected ears (AE) and unaffected ears (UE) [27,29,30].

**Figure 4:**
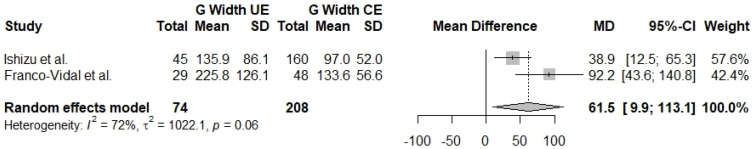
Forest plot comparing G width at 2 kHz between unaffected ears (UE) and control ears (CE) [27,30].

The G width 2 kHz measurement in affected ears compared with control ears was reported in two studies [27,30] with a total of 313 ears (AE *n* = 105, CE *n* = 208). Control ears have a statistically significantly lesser G width of 2 kHz when compared with AE (Mdiff = 93.2, 95% CI: [44.8]–[141.6], I^2^ = 72%). The same measurement comparing affected ears and unaffected ears was reported in three studies [27,29,30], including a total of 216 ears (AE *n* = 124, UE *n* = 92). Unaffected ears have a statistically significant lesser G width of 2 kHz when compared to affected ears (Mdiff = 36.9, 95% CI: [7.4]–[66.5], I^2^ = 0%). Finally, unaffected ears were compared with control ears, and results were reported in two studies [27,30] with a total of 282 ears (UE *n* = 74, control *n* = 208). The results show that control ears have a statistically significant lesser G width of 2 kHz when compared with unaffected ears (Mdiff = 61.5, 95% CI: [9.9]–[113.1], I^2^ = 72%) (Figure 2, Figure 3 and Figure 4).

#### 3.2.2. Resonant Frequency (RF)

**Figure 5:**
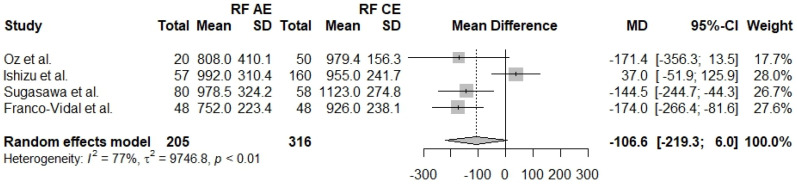
Forest plot comparing RF between affected ears (AE) and control ears (CE) [27,28,30,31].

**Figure 6:**
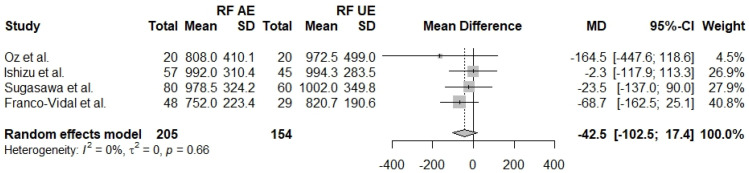
Forest plot comparing RF between affected ears (AE) and unaffected ears (UE) [27,28,30,31].

**Figure 7:**
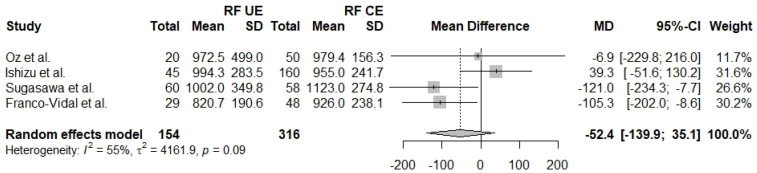
Forest plot comparing RF between unaffected ears (UE) and control ears (CE) [27,28,30,31].

The resonant frequency is the frequency of minimum resistance and maximum amplitude in a vibratory system. A comparison of RF in affected and unaffected ears is reported in five studies [27,28,29,30,31] with a total of 359 ears (AE *n* = 205, UE *n* = 154). The two groups seem to be comparable regarding RF (Mdiff = −42.5, 95% CI: [−102.5]–[17.4], I^2^ = 0.66%). The same measurement between affected ears and control ears was also reported in four studies [27,28,30,31] with a total of 521 ears (AE *n* = 205, CE *n* = 316). The results show no statistically significant differences between the two groups (Mdiff = −106.6, 95% CI: [−219.3]–[6.0], I^2^ = 77%). Four studies [27,28,30,31] directly compare unaffected ears with control ears and report results from a total of 470 ears regarding RF (UE *n* = 154, Control *n* = 316). The results show no statistically significant difference regarding RF in unaffected and control ears (Mdiff = −52.4, 95% CI: [−139.9]–[35.1], I^2^ = 55%) (Figure 5, Figure 6 and Figure 7).

#### 3.2.3. PTA Basic

**Figure 8:**
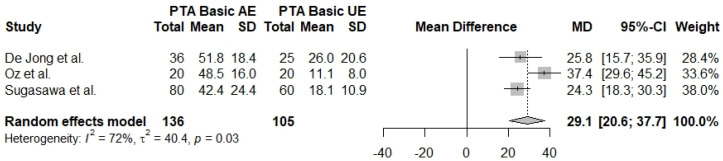
Forest plot comparing PTA basic between affected ears (AE) and unaffected ears (UE) [28,31,32].

The pure tone audiometry average of the lower frequencies measurement comparing affected ears with unaffected ears is reported in three studies [28,31,32] with a total of 241 ears (AE *n* = 136, UE *n* = 105). The results show that PTA basic is statistically significantly greater in affected ears when compared with unaffected ears (Mdiff = 29.1, 95% CI: [20.6]–[37.7], I^2^ = 72%) (Figure 8).

## 4. Discussion

In this meta-analysis, we examine the role of MFT in the diagnosis of MD. To the best of our knowledge, we collected and statistically analyzed all available data from comparative studies using MFT in MD-affected, MD-unaffected, and control ears. Our aim is to provide healthcare specialists with all the available evidence and outcomes of MFT when applied to the diagnosis of MD in clinical practice.

As already mentioned in the introduction, classic tympanometry is a well-described technique in audiology used in the diagnosis of specific middle ear diseases [33]. However, because of the inability of the conventional technique to detect more complicated alterations in the middle ear mechanics following middle ear pathology, a paradigm shift from low-probe tympanometry to MFT has occurred [34]. MFT is an objective, non-invasive, easy, and fast-to-perform technique [34]. Moreover, it is an elaborate sweep-frequency diagnostic tool used to assess the acoustic immittance of the middle ear system, applied in clinical practice during the last decades. Specifically, it elicits a broad spectrum of information from 226 to 2000 Hz, which, although difficult in many cases to interpret, provides an integrated image of the mechanical characteristics of the examined ear [35].

During the last 20 years, an interest in the realization of MFT measurements in patients with inner ear diseases has emerged and relies on the impact of the inner ear pathology on inner ear pressures and subsequently on MFT measurements [7,30]. At the same time, MD is considered to be associated with an increase in production or decrease in absorption of the endolymph, leading to distention of the endolymphatic space or endolymphatic hydrops [36].

The acoustic impedance is an analog of the electrical impedance and represents the resistance of a mechanical system to the flow of acoustic energy through it. The following formula describes the acoustic impedance (Za) of a system:

Za = P/V

where P means sound pressure and V means volume velocity or volume displacement [37].

By utilizing a probe tone frequency that ranges from 226 Hz to 2 kHz, MFT makes it possible to measure both impedance Z and its reciprocal admittance Y based on the pressure exerted in the external auditory meatus canal. Compared to conventional 226 Hz tympanometry, the former is more intricate and involves calculations that consider the phase angle.

The value of the phase angle is crucial in determining the trigonometric breakdown of Y into conductance G (G = Y cos) and susceptance B (B = Y sin). The conductance G, which represents the “real” component of Y, reflects the resistive factors primarily located within the inner ear and reaches its peak at the RF of the middle ear when B = 0. On the other hand, susceptance B is referred to as the “imaginary” component, and it denotes the reactive forces of the middle ear system. The susceptance B is actually the sum of the mass susceptance at the ossicular chain and the susceptance of compliance at the level of the annular ligament and cochlea. At the RF, these two components neutralize each other [15].

At the RF of the middle ear, the values of mass and stiffness are reciprocal and nullify each other. In other words, the phase angle of the middle ear admittance is 0°. When system mass increases, RF decreases. When the stiffness effect dominates the system, RF increases.

MFT in MD could serve to assess perilymph pressure by testing the impedance of the stapes footplate. However, it is uncertain how variations in the pressure of the endolymph that occur in MD affect the pressure of the perilymph, and it can only be hypothesized that changes in the former impact the latter [30].

Measurement of the middle ear’s impedance at different frequencies, varying from low to high, presents high sensitivity in the detection of minor changes in the tympano-ossicular system. This relies on the fact that acoustic immittance measurements at higher frequencies better depict the pathology of the ossicles [35]. Apart from MFT, wideband tympanometry has also been used to evaluate the presence of endolymphatic hydrops in the inner ear. This is a technique similar to MFT used to assess the absorbance of sound energy through the middle ear, namely the acoustic absorbance, at frequencies between 226 and 8000 Hz [38].

The theoretical ability of MFT to distinguish between the contributions of the middle ear and cochlea in admittance allows us to investigate not only tympano-ossicular impairments but also inner ear anomalies. In an experimental study on guinea pigs by Darrouzet et al., a round window fistula induces multiple peak patterns in the Y and G tympanograms and also modifications of the B plot. On the other hand, an increase in cochlear pressure induced by saline injection leads to a widening between the two G peaks at 2 kHz. Thus, admittance, conductance, and susceptance at 2 kHz could represent the effect of the annular ligament and endolymphatic pressure [15].

Significant alterations in the B and G curves at 2 kHz have been reported in humans with sound trauma or MD [39,40]. Additionally, a significant increase in the width of the G curve at 2 kHz has been observed in hemodialyzed patients with chronic kidney disease, suggesting the presence of endolymphatic hydrops or an increased inner ear pressure in this patient category [41]. According to Franco-Vidal et al., changes in the RF and G width of the system could be related to alterations in the inner ear pressure levels [30]. Moreover, an enlarged vestibular aqueduct can present with reduced RF and an air-bone gap, owing to either the third window effect or the presence of endolymphatic hydrops [42].

It appears that at frequencies below 1 kHz, the rigidity of the annular ligament dominates the impedance of the stapes and cochlea, while at frequencies above 1 kHz, it is the mechanical characteristics of the liquids of the cochlea that dominate the system. Thus, it is logical to assume that at 2 kHz, the mass and resistance of the liquids in the cochlea dominate the admittance tympanometry and its components [30].

In this systematic review, we analyze the studies that investigate the possible role of MFT in the diagnosis of MD in humans. Seven studies fulfilled the criteria of our systematic review and meta-analysis. MFT could be an important tool in the diagnosis of MD in the intercritical period but is of no significance during an MD episode [23].

It is of interest that unaffected ears in patients with unilateral MD, when compared to control ears, appear to have a statistically significantly higher G width of 2 kHz, while ears affected with MD have a statistically significantly higher G width of 2 kHz compared to unaffected and control ears [27,29,30]. Thus, G width 2 kHz measurements in unaffected ears are found between those in affected and control ears. These alterations in the MFT of unaffected ears could reveal a subclinical condition and highlight the possibility of bilateral disease even up to ten years following presentation in one ear. The significance of this finding is magnified when clinicians are called to decide on performing a vestibular nerve section or labyrinthectomy. In a study by Paparella and Griebie, bilateral disease occurred in 32% of the patients with MD. Bilateral disease appeared in half of the cases in the first two years [43]. MFT could play a role in diagnosing ears at high risk of MD in patients with unilateral disease.

According to Franco-Vidal et al., 97.9% of affected and 95.8% of control ears presented an ‘M’ shape in the G width 2 kHz. A value of 235 daPa in G width 2 kHz measurement was proposed as a threshold, with values greater considered indicative of MD. Test sensitivity was 56.5% for affected ears and 45.8% for unaffected ears. Only 4.26% of control ears had a positive test. On the other side, the sensitivity of electrocochleography in MD diagnosis is between 57 and 77%, whereas the sensitivity of audiometry with a glycerol test is between 47 and 60% [30]. According to Ishizu et al., however, a threshold of 235 daPa presented a sensitivity of 22.8% and a specificity of 98.8% [27].

Ishizu et al. also reported a significant overlap in the values of G width between the affected and control ears. Additionally, we should not disregard that G width is majorly affected by the middle ear mechanical characteristics, so G width should be regarded as an indicator of MD and not as its sole diagnostic feature. It is proposed that different clinical tools fail to diagnose MD with high accuracy due to the different pathophysiological mechanisms involved. Furthermore, not all types of MD can be diagnosed solely with G width measurements [27].

Moreover, it should be mentioned that no statistically significant differences were found between RF measurements in affected and control ears, and different studies did not all present a reduction in RF. The same applies to comparisons between affected and unaffected ears, as well as between unaffected and control ears. However, the appearance of non-statistical significance between the affected and control ears is marginal, and further studies with larger subject samples should be conducted to delineate the role of RF in the diagnosis of MD. It appears, though, that the intermediate condition of unaffected ears in individuals with unilateral MD cannot be assessed with the use of RF. It should also be kept in mind that other conditions, such as ossicular chain discontinuity and traditional stapedotomy, also decrease RF while increasing G width [44].

According to Sugasawa et al., a measurable RF was reported in 100% of the affected, 95% of the unaffected, and 98.3% of the control ears, and a cutoff value of 875 Hz was proposed [28].

In a study by Oz et al. [31], affected ears at 1 h after the glycerol test showed a statistically significant decrease in RF, which disappeared at 3 h. Thus, the glycerol test in combination with MFT could be studied in the future.

A meta-analysis could not be realized regarding the outcome measure Y width of 2 kHz because of insufficient relative data. According to Yasui et al., the threshold of the Y width of a 2 kHz measurement is 255 daPa. Individuals with values below this limit can be considered normal cases, while individuals with a Y width of 2 kHz above it should be considered positive. A sensitivity of 38% with a Y width of 2 kHz was calculated. No correlation between hearing thresholds and the degree of Y width at 2 kHz was observed [26]. According to Sugasawa et al., 91.4% of control ears, 92.5% of affected ears, and 95% of unaffected ears showed double peaks in Y tympanometry at 2 kHz. This ‘M’ shape in Y tympanometry was reproducible on different days in those patients. The Y tympanometry threshold was set at 237.5 daPa with a sensitivity of 47.3% and a specificity of 86.8% [28]. De Jong et al. reported a percentage of 70.5% in the affected and unaffected ears and 60.1% in the control ears, presenting a double-peak Y tympanogram. In the latter study, a sensitivity of 58.3% and a specificity of 66.3% were reported. A basic disadvantage is that they found a relatively high number of false positive results (56%), principally in the unaffected ears. No correlation was found between the Y width of 2 kHz measurements and hearing thresholds. Importantly, a negative test result has a low probability of ‘hiding’ an affected MD ear [32].

As shown in our meta-analysis, a statistically significant increase in PTA levels can be seen in ears affected by MD [28,31,32]. It has been described that patients with MD present with affected PTA curves not only in the lower frequencies but also in the whole frequency spectrum [45]. Affected and unaffected ears 3 h following glycerol administration also showed a statistically significant reduction in PTA levels [31]. An air–bone gap presenting in the PTA of these patients can be attributed to increased cochlear fluid pressure, causing dampening of the stapes footplate motility [46].

Nevertheless, the current systematic review and meta-analysis present several limitations. Despite the risk of bias assessment performed by the authors, who evaluated 86% of the included studies as being of high quality and 14% as being of fair quality, the results of this meta-analysis should be interpreted with caution as most of the included studies are non-randomized and many factors that confound the results may be present. The heterogeneity between the studies regarding some of the reported outcomes is considered substantial or considerable. Small samples of patients and healthy subjects are examined in relative studies. In addition, the different research teams used different diagnostic criteria in identifying MD. Furthermore, there are inherent differences between medical professionals, patient comorbidities, and middle ear mechanics that can also affect MFT measurements. Finally, the follow-up of the studies was not sufficient to know if certain of the unaffected ears would also develop MD. Despite all the above limitations, the evidence presented in this study is currently the best available evidence to our knowledge.

In general, we would suggest the implementation of MFT, along with history taking, clinical examination, and pure-tone audiometry in the audiology department, as a quick screening tool to assist in the diagnosis of MD. Specifically, in centers where MFT is available, we believe it can provide valuable measurements, not only in the differential diagnosis of middle ear diseases but also in MD [7]. Further assessment should follow if the initial results are indicative of MD.

In conclusion, we found that ears affected by MD show a statistically significant increase in G width at 2 kHz in MFT compared to unaffected ears and control ears. A statistically significant increase in PTA was also observed. On the contrary, there was no statistically significant change in the RF. No significant statistical differences could be exported relatively to the Y width of 2 kHz. Nevertheless, further studies should be conducted to clarify the definitive role of MFT in MD, the mechanism that underlies these measurements, and the diagnostic accuracy of this test.

## Figures and Tables

**Table 1:** Study and patient characteristics.

Author	Year	Study Design	Total Ears Examined	Affected/Unaffected/Control Ears	RF, Mean ± SD	PTA Basic, Mean ± SD	G Width (2 KHz), Mean ± SD	Y Width (2 KHz), Mean ± SD
De Jong et al. [32]	2023	Prospective, not matched	125	AE: 36 UE: 25 CE: 64	N/A	AE: 51.8 ± 18.4 UE: 26 ± 20.6 CE: N/A	N/A	AE: 315.6 ± 70.2 UE: 292.3 ± 98.6 CE: 259.4 ± 60.6
Oz et al. [31]	2019	Prospective, not matched	90	AE: 20 UE:20 CE: 50	AE: 808 ± 410.1 UE: 972.5 ± 499 CE: 979.4 ± 156.3	AE: 48.5 ±16 UE: 11.2 ± 8 CE: N/A	N/A	N/A
Ishizu et al. [27]	2018	Prospective, not matched	262	AE: 57 UE: 45 CE: 160	AE: 992 ± 320.4 UE: 994.3 ± 283.5 CE: 955 ± 241.7	N/A	AE: 166 ± 130.9 UE: 135.9 ± 86.1 CE: 97 ± 52	N/A
Sugawasa et al. [28]	2013	Prospective, not matched	198	AE: 80 UE: 60 CE: 58	AE: 978.5 ± 324.2 UE: 1002 ± 349.8 CE: 1123± 274.8	AE: 42.4 ± 24.4 UE: 18.1 ± 10.9 CE: N/A	N/A	AE: 256.5 ± 126.1 UE:226.2 ± 138.7 CE: 175.4 ± 66.6
Kato et al. [29]	2012	Prospective, not matched	50	AE: 19 UE: 18 CE: 13	AE: 917.4 ± 205.3 UE: 926.9 ± 156.3 CE: N/A	N/A	AE: 169.2 ± 100.3 UE: 107.2 ± 84.1 CE: N/A	N/A
Franco-Vidal et al. [30]	2005	Prospective, not matched	125	AE: 48 UE: 29 CE: 48	AE: 752 ± 223.4 UE: 820.7 ± 190.6 CE: 926 ± 238.1	N/A	AE: 252 ± 120.6 UE: 225.8 ± 126.1 CE: 133.6 ± 56.6	N/A
Yasui et al. [26]	2012	Prospective, not matched	49	AE: 22 UE: N/A CE: 27	N/A	N/A	N/A	AE: 230 ± 15 UE: 179 ± 9 CE: 157 ± 14

Abbreviations: SD: Standard Deviation; AE: Affected Ears; UE: Unaffected Ears; CE: Control Ears; RF: Resonant Frequency; PTA: Pure Tone Audiometry; N/A: Not Available.

**Table 2:** Summary of analysis of outcomes.

Outcomes	*n*	Mean Difference [95% CI]	Heterogeneity	
			*I^2^*	*p*
**G Width 2 kHz (AE–UE)**	3	36.9 [7.4; 66.5]	0%	0.63
**G Width 2 kHz (AE–CE)**	2	93.2 [44.8; 141.6]	72%	0.06
**G Width 2 kHz (UE–CE)**	2	61.5 [9.9; 113.1]	72%	0.06
**PTA Basic (AE–UE)**	3	29.1 [20.6; 37.7]	72%	0.03
**RF (AE–UE)**	4	−42.5 [−102.5; 17.4]	0%	0.66
**RF (AE–CE)**	4	−106.6 [−219.3; 6.0]	77%	<0.01
**RF (UE–CE)**	5	−52.4 [−139.9; 35.1]	55%	0.09

Abbreviations: AE = affected ears; UE = unaffected ears; CE = control ears; CI = Confidence Intervals.

## Data Availability

Data are contained within the article.

## Supplementary Materials

The following supporting information can be downloaded at: https://www.mdpi.com/article/10.3390/jcm13051476/s1, Table S1: Quality of evidence assessment using Newcastle-Ottawa scale.
