# Supplementary material for: Can Multifrequency Tympanometry Be Used in the Diagnosis of Meniere’s Disease? A Systematic Review and Meta-Analysis
Source: J Clin Med. 2024 Mar 4;13(5):1476. doi: 10.3390/jcm13051476 (PMC10932169; doi:10.3390/jcm13051476)
Supplement: Supplementary file 1 [file jcm-13-01476-s001.zip › jcm-2830666-supplementary.pdf]

Table S1. Quality of evidence assessment using Newcastle-Ottawa scale.

Author/ Year	SELECTION				COMPARABILITY	EXPOSURE			Total	Quality
	Is the case definition adequate?	Representativeness of the cases	Selection of controls	Definition of controls	Comparability of cases and controls on the basis of the design or analysis	Assessment of outcome	Same method of ascertainment for cases and controls	Non-Response rate		
De Jong et al. 2023	0	0	0	1	1	1	1	1	5/9	Fair
Oz et al. 2019	1	1	0	1	1	1	1	1	8/9	High
Ishizu et al. 2018	1	0	0	1	2	1	1	1	7/9	High
Sugasawa et al. 2013	1	0	1	1	2	1	1	1	8/9	High
Kato et al. 2012	1	0	0	1	1	1	1	1	7/9	High
Franco-Vidal et al. 2005	1	1	0	1	1	1	1	1	7/9	High
Yasui et al. 2012	1	1	0	1	1	1	1	1	7/9	High
